# Arhovin in the Treatment of Gonorrhea

**Published:** 1907-04

**Authors:** 


### Arhovin in the Treatment of Gonorrhea

Dr. Hernfeld, Emeritus Assistant of the Allgemeine Krankenhaus at Vienna, writes (*Therapie der Gege wart,* April, 1906,) regarding the experience of his division with the internal therapy of gonorrhea by arhovin. This chemical combination of diphenylamine and esterified thymyl-benzoic acid is absorbed a short time after ingestion, as the green reaction of the urine with Fe Cl shows. Though best administered in capsule form, it is also readily taken mixed with sugar.

Arhovin is split up in the gastrointestinal tract, its constituents passing into the urine. That excretion thus acts as a disinfectant and specifically gonocidal in the passages which are flushed by it, and besides its acidity is enhanced. In a whole series of cases in which no other medicinal or local treatment was used, arhovin indubitably effected a subsidence of the acute inflammatory manifestations of gonorrhea, such as swellings of the orifice, tenderness of the urethra to pressure, painful micturition; also in acute posterior urethritis there was also a rapid retrogression of the dysuric symptoms, tenesmus, chordee, and the like.

In the last six months about 40 cases were treated with arhovin in his division. In the first group there were fresh gonorrheas hitherto not treated, with more or less severe inflammatory symptoms. Among 25 acute gonorrheas there were 2 with epididymitis and 5 with considerable cystitis or posterior urethritis. All these patients bore the remedy very well; when it was alternated with sandalwood oil, the patients often complained of eructations and digestive disturbances.

In a series of prostatitis cases 6 arhovin capsules were given daily for three or four weeks after the cessation of the first irritative symptoms, with good effect. Combined with vesical lavage, it was equally successful in obstinate cystitis. Occasionally no effect was seen at first, as in three epididymitis cases; but upon continued use there was retrogression of the badly inflamed epididymis; and in a short time the strongly purulent secretion was in all cases materially diminished and in two entirely inhibited.

The favorable course of this severe complication, though all patients were ambulatory, shows that these results are not accidental. Unlike the balsams, arhovin does not aggravate the inflammation, but on the contrary is anti-inflammatory or at least indifferent. By inhibiting gonococcal growth on the mucosæ, arhovin prevents a further invasion of the urethra by inflammation-products, toxins and bacteria, so that the protective power of the testicular parenchymal cells is able to overcome the toxins already present.

The second group consists of cases of sub-chronic, protracted gonorrhea in which there was a strong and purulent gonococcal secretion. Thus we had a case of severe urethritis of eight months' standing, with deep mucosal infiltrations in the anterior and posterior urethra. Janet's treatment was impossible on account of the thickening of the mucosa; and after each cauterisation by urethral sound there was such an intense irritation with secretion that the patient could not be treated at all for several days; but when arhovin was also used, the sounds were well borne. Under this combined treatment the secretion lessened to a minimum, so that the results were excellent. Similar observations were made in two other cases with regard to the eminent secretion-limiting effect of arhovin in the posterior urethra and the region of the vesical neck, in chronic gonorrhea with involvement of the prostate; in these there is always increase of the secretion and urinary cloudiness after local treatment, until arhovin internally was also given. Then the characteristic favorable effects noted elsewhere followed.

In three other chronic cases, anterior and posterior, with inflammatory symptoms, arhovin was advantageously used after other internal remedies had failed, and in a series of phosphaturia cases it seemed to clear the urine. In severe cystitis, which had for some time received silver nitrate irrigations, the use of arhovin was followed in a few days by clearing of the very cloudy urine.

